# Demographic, diagnostic and therapeutic characteristics of autosomal dominant polycystic kidney disease in Ghana

**DOI:** 10.1186/s12882-021-02336-8

**Published:** 2021-04-28

**Authors:** Perditer Okyere, Richard K.D. Ephraim, Isaac Okyere, Joseph Attakorah, Dorcas Serwaa, Grace Essuman, Albert Abaka-Yawson, Prince Adoba

**Affiliations:** 1 Department of Medicine, School of Medicine and Dentistry, College of Health Sciences, Kwame Nkrumah University of Science and Technology, Kumasi, Ghana; 2 Department of Medical Laboratory Science, School of Allied Health Sciences, University of Cape Coast, Cape Coast, Ghana; 3 Renal Research Initiative , Cape Coast, Ghana; 4 Department of Surgery, School of Medicine and Dentistry, College of Health Sciences, Kwame Nkrumah University of Science and Technology, Kumasi, Ghana; 5 Department of Pharmacy Practice, Faculty of Pharmacy, College of Health Sciences, Kwame Nkrumah University of Science and Technology, Kumasi, Ghana; 6 Department of Obstetrics and Gynecology, College of Medicine, Institute of Life and Earth Sciences, Pan African University, University of Ibadan, Ibadan, Nigeria; 7 Department of Medical Laboratory Science, School of Allied Health Sciences, University of Health and Allied Sciences, Ho, Ghana; 8 Trauma and Specialist Hospital, Ghana Health Service, Winneba, Ghana

**Keywords:** Autosomal dominant polycystic kidney disease, Chronic kidney disease, Demographic characteristics, Clinical features, Biochemical derangements

## Abstract

**Background:**

Autosomal Dominant Polycystic Kidney Disease (ADPKD) is the commonest of the hereditary kidney diseases and mostly ensues in utero with signs delayed until after several decades. This study assessed the demographic, diagnostic (clinical and biochemical features) and therapeutic patterns among ADPKD patients who attended the nephrology unit of Komfo Anokye Teaching Hospital (KATH) from 2007 to 2018.

**Methods:**

This cross-sectional retrospective analysis of ADPKD patient records was conducted at the nephrology unit of KATH in October 2020. The records of 82 ADPKD was used for this study. Demographic, clinical, biochemical, ultrasonographic and therapeutic data was obtained, organized and analyzed with Statistical Package for the Social Sciences (SPSS).

**Results:**

ADPKD was most prevalent in people within the ages of 31–40 years (25.6 %), with a male (52.4 %) preponderance. The most common clinical features presented were flank pain (30.5 %) and bipedal swelling (18.3 %). Hypertension (42.7 %), urinary tract infections (UTIs) (19.5 %), and anemia (13.4 %) were the most common complications reported. Average level of HDL-c was higher in females (1.7) than in males (1.2) (*p* = 0.001). Hematuria (34 %) and proteinuria (66 %) were among the biochemical derangements presented. About 81.7 % had CKD at diagnosis with the majority in stages 1 (27.0 %), 3(23.2 %) and 5 (20.3 %). Poor corticomedullary differentiation was observed in 90.2 % of participants and increased echogenicity was observed in 89.0 % of the participants. Estimated GFR (eGFR) correlated positively with echotexture (*r* = 0.320, *p* = 0.005) and negatively with CMD (*r*= -0.303, *p* = 0.008). About 95.1 % of patients were on conservative therapy including: 73.2 %, 52.4 %, 22.0 %, 13.4 %, 8.5 % on Irebesartan/Lisinopril, Nifecard XL, Hydralazine, Methyldopa and Bisoprolol respectively for hypertension; 26.8 and 3.7 % on Gliclazide and Metformin respectively for Type 2 diabetes mellitus; 25.6 %, 24.4 and 18.3 % on CaCO_3_, fersolate and folic acid respectively as nutrient supplements with 4.9 % of participants on renal replacement therapy (RRT).

**Conclusions:**

ADPKD occurs in people aged ≥ 31 years with a higher male preponderance. Clinical features include flank and abdominal pain, bipedal swelling, headache, amongst others. Uremia, hematuria, proteinuria, decreased eGFR, were the common biochemical derangements reported with higher severity detected in men. The therapeutic interventions mostly involved conservative therapy to manage symptoms and other comorbid conditions and rarely renal replacement therapy (RRT).

## Background

Autosomal Dominant Polycystic Kidney Disease (ADPKD) is the most common form of hereditary kidney disease that mostly manifests during adulthood [1]. Characterized by the formation and growth of multiple cysts, ADPKD distorts kidney structure and function and ultimately results in End Stage Kidney Damage (ESRD) [2]. The genetic basis of the condition is a mutation in the polycystin genes (PKD1 and PKD 2) and rarely, the recently detected GANAB [3] and PMM2 [4] genes. Disease course is often associated with other extra-renal disease characteristics including cyst formation in different organs such as the spleen, liver, pancreas, etc.; abdominal wall hernias; cardiovascular abnormalities among others which contribute to disease morbidity [5]. It is a lifelong progressive condition and until recently, there was no ADPKD specific treatment [6, 7].

ADPKD is the fourth highest cause of CKD in Ghana [8] and accounts for about 5–10 % of ESRD patients worldwide[9]. In Europe, it is noted as the fourth kidney disease diagnosis that requires renal replacement therapy (RRT), with an estimate of about one tenth of all RRT patients having ADPKD [6].

Diagnosis of the ADPKD is based on family history, clinical presentations and radiological imaging. A positive family history; presence of clinical features such as: hematuria, back pain etc. and radiologic proof of bilateral fluid-filled renal cysts confirms the disease [9]. In the developed world, advanced genetic testing is also employed in the screening and diagnosis of ADPKD [10].

The diagnostic and therapeutic characteristics of the disease need to be clearly defined especially in our context. In Africa, only few studies have been conducted on ADPKD making information on ADPKD specific for Africans especially Ghanaians very scarce. With this scarcity of data, we are compelled to rely on information from advanced countries for the diagnosis and management of our patients. Differences in geographical location, genetic predispositions, climatic conditions, economic and infrastructural resources, sometime makes this inappropriate. It is therefore necessary to provide population-specific information regarding ADPKD especially in Ghana. In light of the above we sought to assess the demographic, diagnostic (clinical and biochemical) and therapeutic patterns among ADPKD patients who attended the nephrology unit of KATH, Ghana.

## Methodology

### Study site/ study design

This cross-sectional retrospective study of patient records was conducted at the nephrology unit of Komfo Anokye Teaching Hospital (KATH) during the period of 2007 to 2018. KATH is the second largest hospital in Ghana with 1000 bed capacity and a well-resourced nephrology unit. The nephrology clinic is an adult (for ages 18 years and above) nephrology clinic, with services sometimes extending to patients as young as 14 years of age.

### Eligibility criteria

Records of patients diagnosed with ADPKD after clinical, imaging and biochemical testing within the period under review was used. Records of participants with other renal diseases and those with incomplete data were excluded.

### Participants/collection of retrospective data

Data of 82 ADPKD patients (male/female) was manually collected from the records of the nephrology unit. The period of data collection was from 2007 to 2018. Demographic information of each participant including gender, age, geographical location and occupation was collected. Clinical information obtained included type of renal disease presented, presence or absence of hypertension, other non-renal diseases presented, results of biochemical tests and treatment administered to the patient and ultrasound results.

### Ethical consideration

Ethical clearance for the study was obtained from the research and development unit of KATH. Data used was obtained in adherence to the principles of the declaration of Helsinki and local regulatory requirements.

### Data analysis

Data was analyzed using Statistical Package for the Social Sciences (SPSS), version 22.0. Test for normality was performed with box plot, kurtosis and Kolmogorov-Smirnoff test. Descriptive summary statistics such as frequencies, percentages and bar charts were presented as appropriate. Parametric data were presented as means ± standard deviation and median (interquartile ranges) for non-parametric data. The Student’s T test or the Mann–Whitney U test was used appropriately to test the descriptive statistics for continuous variables and the Chi square test for categorical variable was used whenever applicable. Relationships between clinical parameters were assessed with Pearson and Spearman rank tests. The statistical significance of variables were set at *p* value of < 0.05 and 95 % confidence level.

## Results

A total of 82 patients fulfilled the inclusion criteria of ADPKD. The mean ages of the patients at the time of diagnosis was 43.83 ± 15.71 years with an average range of 15–80 years and the peak ages of ADPKD occurrence was between 31 and 40 years (25.6 %) and 51–60 years (20.7 %). Males were 42 (52.4 %) and 39 (47.6 %) females. Regarding their occupational status, 35(42.7 %) were traders followed by unemployed, 14 (17.1 %) (Table 1).

**Table 1:** Sociodemographic characteristics of the study participants

Characteristics	Number (N)	Percentage (%)
**Age (years) Min = 15 Max= 80 Mean= 43.83 ± 15.71**		
15–20	7	8.5
21–30	10	12.2
31–40	21	25.6
41–50	13	15.9
51–60	17	20.7
> 60	14	17.1
**Gender**		
Male	43	52.4
Female	39	47.6
**Occupation**		
unemployed	14	17.1
students	6	7.3
Civil servants	6	7.3
Traders	35	42.7
Farmers	13	15.9
Others	8	9.8

The commonest mode of clinical presentations were flank pain (30.5 %), bipedal swelling (18.3 %) and headache (9.8 %). At the time of presentation, exactly half (50 %) of the patients were comorbid with hypertension, 7 (8.5%) had both hypertension and diabetes mellitus and 33 (40.2 %) presented with no comorbid condition. Sixty (73.2 %) patients were on Irbesartan/Lisinopril; 43 (52.4 %) were on Nifecard XL, 22 (26.8 %) on Gliclazide and 21 (25.6 %) on CaCO_3_ (Table 2).

**Table 2:** Presenting complaints, co-morbidity and therapeutic characteristics of participants

Various presenting complaints		
	Number of patients	%
**Presenting complaints**		
Flank Pain	25	30.5
Bipedal Swelling	15	18.3
Headache	8	9.8
Abdominal Pain	7	8.5
Abdominal Distension	6	7.3
Easy Fatigability	5	6.1
Dizziness	4	4.9
Breathlessness	2	2.4
Early Morning Facial Puffiness	2	2.4
General Body Weakness	2	2.4
Vomiting	2	2.4
Hematuria	2	2.4
Palpitation	1	1.2
Polyuria	1	1.2
**Co-morbid at the time of presentation**		
No co-morbidity	33	40.2
Hypertension	41	50.0
Hypertension with diabetes	7	8.5
Prostatic Hypertrophy	1	1.2
**Therapeutic characteristics at the time of presentation**		
Irbesartan/Lisinopril	60	73.2
NifecardXL	43	52.4
Gliclazide	22	26.8
CaCO_3_	21	25.6
Fersolate	20	24.4
Lasix	20	24.4
Hydralazine	18	22.0
Folic acid	15	18.3
EPO	12	14.6
Methyldopa	11	13.4
Statin	10	12.2
NaHCO_3_	10	12.2
Bisoprolol	7	8.5
Aspirin	3	3.7
Metformin	3	3.7
Ranitidine	1	1.2

EPO: erythropoietin, CaCO_3_: calcium carbonate, NaHCO_3_: sodium bicarbonate

The baseline clinical laboratory findings are summarized in Table 3. The mean systolic pressure was 140.8 ± 26.9 mm Hg and mean diastolic pressure was 78.6 ± 18.1 mm Hg with a mean haemoglobin of 10.3 ± 3.0 (g/dl). The median serum potassium and urea were 4.4 [4.0-4.9] (mmol/L) and 5.7 [3.8–15.2] (mmol/L) respectively. Their median creatinine level was 167.0 [93.7–354.0] µmol/L and their overall median rate of estimated glomerular filtration rate was 45.0 [17.0–96.0] ml/min/1.73m^2^. T-test and Mann-Whitney U test analysis revealed that there was a statistically significant difference between gender and haemoglobin (*p* = 0.038), serum potassium (0.026), urea (0.002) and HDLc (*p* = 0.001). Exactly 33/50 (66.0 %) and 17/50 (34.0 %) of the patients had protein and blood in their urine samples respectively.

**Table 3:** Clinical and Laboratory investigation results in participants

Baseline parameters	All Subjects	Male	Female	p
	mean ± SD	mean ± SD	mean ± SD	
SBP(mmHg)	140.8 ± 26.9	138.6 ± 26.8	142.7 ± 27.2	0.494
DBP (mmHg)	78.6 ± 18.1	86.3 ± 14.9	81.1 ± 13.5	**0.038**
Haemoglobin	10.3 ± 3.0	11.0 ± 3.2	9.6 ± 2.7	0.071
	Median [IQR]	Median [IQR]	Median [IQR]	
Sodium	138.0 [136.0-141.0]	138.0 [136.0-142.0]	138.5 [137.0-141.0]	0.962
Potassium	4.4 [4.0-4.9]	4.7 [4.1–5.3]	4.2 [3.9–4.7]	**0.026**
Urea	5.7 [3.8–15.2]	8.0 [4.3–20.3]	4.3 [3.0-8.4]	**0.002**
Creatinine	167.0 [93.7–354.0]	180.0 [117.6–364.0]	129.0 [66.5–244.0]	0.067
eGFR	45.0 [17.0–96.0]	38.5 [14.6–71.5]	51.0 [23.0-114.5]	0.287
MCV	81.1 [74.4–86.4]	78.7 [74.1–86.2]	81.8 [74.4–86.4]	0.702
MCH	27.7 [25.5–29.5]	27.7 [25.5–29.7]	27.8 [24.3–29.3]	0.409
White Blood Count	5.1 [4.4–6.5]	5.5 [4.6–6.6]	4.9 [4.4–6.5]	0.323
Triglycerides	1.1 [0.8–1.4]	1.0 [0.7–1.4]	1.1 [0.9–1.4]	0.650
LDL-c	3.0 [2.3–4.2]	2.9 [2.3-4.0]	3.0 [2.3–4.2]	0.762
HDL-c	1.3 [1.2–1.8]	1.2 [0.9–1.3]	1.7 [1.3–1.9]	**0.001**
VLDL-c	0.5 [0.4–0.6]	0.5 [0.3–0.6]	0.5 [0.4–0.6]	0.879
Cholesterol	5.0 [4.1-5.0]	4.4 [4.0–6.0]	5.4 [4.1–6.1]	0.524
	N (%)	N (%)	N (%)	
**Urine Protein**				
Positive	33/50 (66.0)	15/22 (68.2)	18/28 (64.3)	0.773
Negative	17/50 (34.0)	7/22 (31.8)	10/28 (35.7)	
**Urine Blood**				
Positive	17/50 (34.0)	8/22 (36.4)	9/28 (32.1)	0.875
Negative	33/50 (66.0)	14/22 (63.6)	19/28 (67.9)	

*SBP* systolic blood pressure, *DBP* diastolic blood pressure, *LDL-c* low-density lipoprotein cholesterol, *HDL-c* high-density lipoprotein cholesterol, *VLDL-c* very low density lipoprotein cholesterol, *MCV* mean corpuscular volume, *MCH* mean corpuscular hemoglobin

Of the 82 ADPKD patients, 67(81.7 %) had stage 1 to 4 chronic kidney disease (CKD), 13(15.9 %) had stage 5(end-stage renal disease - ESRD) and 2(2.4 %) had acute kidney injury (AKI). Twenty participants (27.0 %) presented with stage I, 19 (23.2 %) with stage II and 15 (20.3 %) with stage V CKD. Seventy-eight (95.1 %) of the patients were on medication and conservative treatments while 4(4.9 %) were dialysis dependent at the time of study. Hypertension (42.7 %) and urinary tract infections (19.5 %) were the most common complications. A total of 51 (62.2 %) of the ADPKD patients registered with the hospital were lost to follow-up during the study period, 1(1.2 %) discharged and 2 (2.4 %) patients died following diagnosis (Table 4).

**Table 4:** Diagnosis, staging, treatment and complications in participants

Diagnosis		
	Number of patients	%
CKD	67	81.7
ESRD	13	15.9
AKI	2	2.4
**Staging (*n* = 74)**		
I	20	27.0
II	6	8.1
III	19	23.2
IV	14	18.9
V	15	20.3
**Treatment**		
Medication/Conservative	78	95.1
Dialysis	4	4.9
**Complications**		
No Complications	10	12.2
Hypertension	35	42.7
UTI	16	19.5
Anaemia	11	13.4
Uremic Gastritis	6	7.3
Acute Kidney Injury	2	2.4
Epigastric Hernia	1	1.2
Hyperkalaemia	1	1.2
Fluid Overload	1	1.2
**Follow-Ups**		
Lost to Follow-Up	51	62.2
Alive	28	34.1
Discharged	1	1.2
Dead	2	2.4

**Fig. 1:**
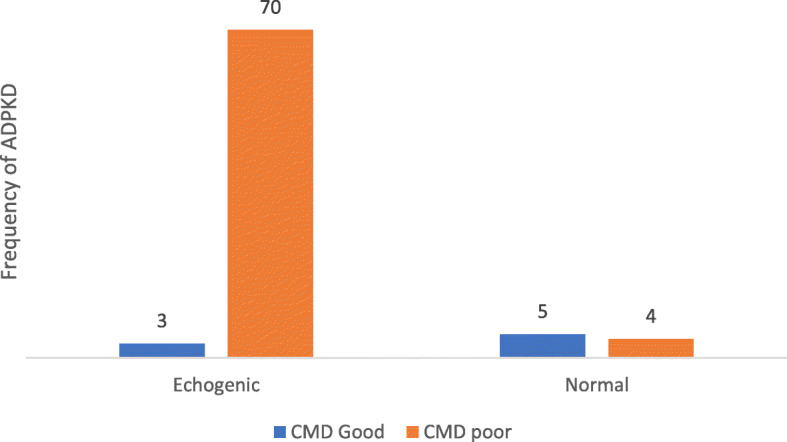
Association between Echotexture and Corticomedullary Differentiation

All the 82 patients had ultrasound examination and length pole-pole (LPP) was 15.56 ± 2.88 cm for the right and 15.47 ± 2.90 cm for the left kidney. Pole width (PW) on the right side was 8.18 ± 2.36 cm slightly less than 8.23 ± 2.00 cm on the left side (Table 5). About 74 (90.2 %) had poor corticomedullary differentiation (CMD) while only 8(9.8) had good CMD. Exactly 89.0 % had increased echogenicity and 9(11.0 %) had normal echotexture (Table 1). Poor corticomedullary differentiation (CMD) was associated with increased echogenicity (*p* < 0.001) (Fig. 1).

**Table 5:** Kidney dimensions as depicted by ultrasonography

Kidney dimensions	All subjects	Male	Female
	mean ± SD	mean ± SD	mean ± SD
Right Kidney			
LPP	15.56 ± 2.88	15.63 ± 2.53	15.49 ± 3.20
PW	8.18 ± 2.36	8.65 ± 2.30	7.76 ± 2.35
Left Kidney			
LPP	15.47 ± 2.90	15.56 ± 2.50	15.39 ± 3.25
PW	8.23 ± 2.00	8.52 ± 1.92	7.97 ± 2.06

*Spearman’s rho

*LPP* pole-to-pole kidney length, *PW* pole width

Correlation analysis revealed that estimated glomerular filtration rate was significantly associated with hemoglobin (Pearson correlation coefficient (*r*) = 0.328, *p* = 0.008), serum potassium (*r*=-0.351, *p* = 0.009), serum urea (*r*=-0.599, *p* < 0.001) and white blood cell count (*r* = 0.438, *p* < 0.001). Also, eGFR negatively correlated with urine protein (Spearman rank correlation coefficient (*r*)= -0.329, *p* = 0.020) and urine blood (*r*= -0.453, *p* = 0.001). Glomerular filtration rate negatively correlated with corticomedullary differentiation (CMD) (*r*= -0.303, *p* = 0.008) and positively correlated with echotexture (*r* = 0.320, *p* = 0.005) (Table 5).

## Discussion

We assessed the demographic, diagnostic and therapeutic characteristics of ADPKD patients in Nephrology clinic at KATH, Kumasi. In line with earlier studies, we found that the disease mostly presented in patients aged 31 years and above, with the highest occurrence within ages 31 to 40 years [1, 11, 12]. This is consistent with existent literature that ADPKD often occurs in adults over 30 years of age [2, 13, 14]. Similar to the studies of Arogundade et al., [15] and Chijioke et al., [16] we detected a male preponderance in disease incidents.

In accordance with Hajji et al., [1], the drugs reported in this study are designed to manage the symptoms, complications and comorbidities associated with the disease. According to Pei et al., [17], the development of hypertension indicates disease progression and must be managed ideally with blood pressure target of 130/80 mm/Hg. It is not surprising that most of our study participants presented with hypertension. The activation of RAAS and sympathetic nervous system are the predominant causes of hypertension in ADPKD. Left ventricular hypertrophy (LVH) also causes high BP and increases cardiovascular risk in this condition [1]. Therefore, early detection and treatment of hypertension could hinder cyst development and cardiovascular complications [18]. Angiotensin converting enzyme inhibitors (ACEI)s and Angiotensin receptor blockers (ARBs) are the preferred medications used in the management of hypertension in ADPKD because they have been proven beneficial to: control cardiac dysfunction; decrease LVH and end organ damage; and reduce mortality[14]. Hence it is not surprising that most of our participants were on Lisinopril and Irbesartan. Again, majority of our participants (95.1 %) were on conservative therapy with very few (4.9 %) on dialysis. Usually in CKD, patients are managed for as long as possible and hemodialysis – usually the last resort – is started when GFR has receded to < 15 mL/min [19].

Bipedal swelling and flank pain are common symptoms reported in patients with ADPKD. In consonance with the study of Mandal et al., [20], we observed bipedal swelling and flank pain among our participants. Bipedal swelling usually occurs due to the lower serum oncotic pressure from the nephrotic proteinuric range characteristic of kidney disease whereas flank pain results directly and indirectly from cyst formation and enlargement on kidneys and sometimes, nearby organs such as the liver [21]. Flank pain was reported by a rather lower percentage (30.5 %) of our participants than that (68.3 %) reported by Arogundade et al., [15], most likely due to our larger sample size. UTIs, were the second highest complication (19.5 %) at diagnosis. Other studies reported much higher percentages with female predominance[15, 22, 23].

With about 97.6 % of our participants having CKD and 15.9 % of them already in end stage, it is typical that anemia was reported in (13.5 %) although this was rather a low perecentage. Some of these patients may have polycythemia resulting from cystic production of erythropoietin hence preventing anemia progression. Most of the patients seem to have normal potassium levels although the levels in males were significantly higher than in females. Typical of CKD and in accordance with the findings of Helal et al., [24] and Arogundade et al., [15] urea and creatinine levels were high for most of the participants. With our study, urea was particularly higher in males than in females. These findings concede with the fact that disease progression is suggested to be faster in men than in women [25]. Others attribute hormonal differences – contributing to disease pathogenesis – to the better prognosis seen in men than in women [26]. With HDL-c levels significantly lower in males than in females, we can infer that cardiovascular risk is higher for males than for females in our study.

**Fig. 2:**
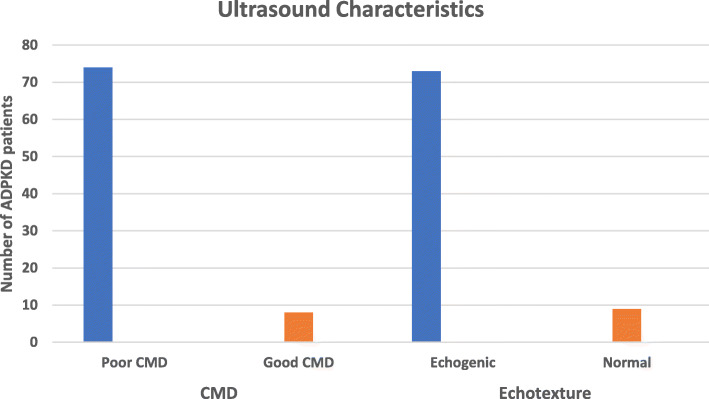
Ultrasound characteristics of the patients with ADPKD

From the correlation analysis, eGFR was negatively correlated with CMD and with 97.6 % of our participants having CKD, it is normal to have 90.2 % of them showing poor CMD upon imaging (Fig. 2). Loss of CMD associated with decreasing eGFR, is characteristic of renal insufficiency resulting from edema, renal atrophy, renal scarring, etc.[27, 28]. The positive correlation between eGFR and echogenicity we found is expected, since increased kidney echogenicity usually signifies an underlying kidney disease. Similar to the findings of Cristea et al., [29], kidney size (defined by the LPP) was significantly high (averagely 15.56 cm for right kidney and 15.47 cm for left - Table 5), most likely due to multiple cyst formation and enlargement in the kidney.

As observed in our study (Table 6), proteinuria and hematuria are common observations in ADPKD [30]. Whereas hematuria in ADPKD mostly occurs either due to hemorrhage into cysts or cyst rapture into collecting ducts, proteinuria occurs due to impaired reabsorption of proteins by cyst-lined tubular epithelia [31] but is not usually observed in the early stages [32].

**Table 6:** Correlation between serum glomerular filtration rate (eGFR) and clinical laboratory parameters

Parameter	eGFR	
	r	p
Haemoglobin	0.328	0.008
Potassium	-0.351	0.009
Urea (mg/Dl)	-0.599	< 0.001
White Blood Count	0.438	< 0.001
Urine protein	-0.329	0.020*
Urine blood	-0.453	0.001*
CMD	-0.303	0.008*
ECT	0.320	0.005*

*CMD* corticomedullary differentiation, *ECT* echogenecity

## Limitations

Our study has the following limitations. First, data used was collected from one nephrology clinic and follow up information was not documented. Second, most of the study participants were lost to follow up, hence survival analysis could not be well performed,although, with the data we had, only two of our participants died due to causes not known to us.Third, there is no such data present in the country, hence we cannot conclusively draw comparative inferences on patient outcomes. Fourth, due to the inability to detect ADPKD early in disease course [5], milder forms of the disease may have been excluded.

## Conclusions and recommendations

In Ghana ADPKD mostly occurs in people of age 31 years and above with a higher male preponderance. Clinical features include flank and abdominal pain, bipedal swelling, headache, amongst others. Uremia, hematuria, proteinuria and decreased eGFR are some of the biochemical derangements presented by patients with this condition. Hypertension turned out to be the most common comorbidity as well as complication associated with ADPKD with proteinuria, hematuria, UTIs, anemia amongst others developing as disease progresses. The therapeutic interventions mostly involve conservative therapy to manage symptoms and other comorbid conditions and rarely renal replacement therapy (RRT). Early diagnosis of the disease should be targeted by engaging more advanced ultrasonography. Also, molecular diagnosis may be employed for early diagnosis in high-risk individuals such as those with a family history of the disease. Future researchers may explore multicenter studies and engage proper monitoring regimens to analyze disease outcomes in our part of the world,

## Data Availability

The Datasets used for this study are not publicly available because they contain information that could compromise the privacy of the study participants but are available from the corresponding author on reasonable request.

## Declarations

The authors give consent for the publication of text material including all identifiable details within this manuscript to be published in BMC Nephrology journal.
